# Fe_3_O_4_ Nanoparticles Functionalized with Polymer Ligand for T_1_-Weighted MRI In Vitro and In Vivo

**DOI:** 10.3390/polym11050882

**Published:** 2019-05-14

**Authors:** Chenyang Xiang, Xin Zhong, Weitao Yang, Muhammad Irfan Majeed, Jun Wang, Jiani Yu, Jinming Hu, Zushun Xu, Bien Tan, Bingbo Zhang, Wei Yan

**Affiliations:** 1 Hubei Collaborative Innovation Center for Advanced Organic Chemical Materials, Ministry of Education, Key Laboratory of Green Preparation and Application for Functional Materials, Hubei Key Laboratory of Polymer Materials, School of Materials Science & Engineering, Hubei University, Wuhan 430062, China; xiang13797531564@163.com (C.X.); xingzh@hotmail.com (X.Z.); zushunxu@hubu.edu.cn (Z.X.); 2 The Institute for Biomedical Engineering & Nano Science, Tongji University School of Medicine, Shanghai 200443, China; weitaoyang@tongji.edu.cn (W.Y.); wangjun016@tongji.edu.cn (J.W.); 1532091@tongji.edu.cn (J.Y.); 3 School of Chemistry and Chemical Engineering, Huazhong University of Science and Technology, Wuhan 430074, China; irfanmajeed2003@gmail.com; 4 CAS Key Laboratory of Soft Matter Chemistry, Department of Polymer Science and Engineering, University of Science and Technology of China, Anhui 230026, China; jmhu@ustc.edu.cn

**Keywords:** MR imaging, Fe_3_O_4_ nanoparticles, ultra-small, low toxicity, T_1_ contrast agents

## Abstract

Magnetic resonance imaging (MRI) has gained wide interest in early accurate diagnoses due to the high resolution and low toxicity of magnetic nanoparticles. In order to develop potential alternatives of toxic Gd- or Mn-based chelating agents, we report the synthesis of water soluble ultra-small Fe_3_O_4_ nanoparticles by a modified co-precipitation method as T_1_-weighted positive contrast agents. The magnetic iron oxide nanoparticles (MIONs) were functionalized by polymer ligand dodecanthiol-polymethacrylic acid (DDT-PMAA) to enhance their colloidal stability. These MIONs have high longitudinal relaxivity (*r*_1_ = 8.18 mM^−1^·S^−1^) and exhibited good results in the in vitro and in vivo MR imaging. No toxicity was observed in cytotoxicity assay and histology toxicity analysis. The MIONs@DDT-PMAA(magnetic iron oxide nanoparticles @ dodecanthiol-polymethacrylic acid) present great potential as positive contrast agents for tumor diagnosis.

## 1. Introduction

Magnetic resonance imaging (MRI) has become the most widely used diagnostic tool because of its high spatial resolution, unlimited tissue penetration, and low radioactive damage [1,2,3,4,5]. MR contrast agents are a series of contrast media that can significantly improve the resolution and specificity of MRI by reducing the relaxation times in the local tissue of the body [6,7,8,9]. Iron oxide nanoparticles have been widely studied because of their biocompatibility, targeted imaging in vivo, and magnetic separation. These iron nanoparticles exhibited great negative contrast effects and favorable low toxicity of Fe ions [10,11,12,13]. In recent years, superparamagnetic iron nanoparticles have been found to shorten T_2_ relaxation times as the most negative contrast agents have been used in clinic, such as ferumoxsil and ferumoxyltol [14,15,16,17]. However, the dark signals provided by T_2_ contrast agents can be confused with other hypointense regions such as air, metal deposition, and blood clots [18,19,20]. In addition to the dark signals, medical workers have also observed bright signals. In the biomedical field, T_1_ contrast agents are mainly paramagnetic Gd- or Mn-based agents [20]. Nevertheless, the damage caused by the paramagnetic Gd in patients’ kidneys are still of great concern and Mn-based chelating agents have also been criticized for possible neurotoxicity [21,22,23,24,25,26]. Therefore, there is a dire need to develop new positive contrast agents that are biocompatible and non-toxic.

Roca et al. reported that the magnetic properties of iron nanoparticles depend enormously on their size [27]. The coupled magnetic moment of iron nanoparticles decreases rapidly as their size decreases, which can greatly restrain the T_2_ contrast effect and conversely enhance the T_1_ contrast effect [28,29,30,31,32]. Generally, ultra-small iron nanoparticles (˂5 nm) can be used as T_1_ contrast agents [31,33]. 

Among the techniques used for the synthesis of iron oxide nanoparticles, thermal decomposition and co-precipitation are the most studied, but both of these methods are generally associated with some drawbacks. The thermal decomposition method can be used to obtain uniform and ultra-small nanoparticles, but their poor water solubility limits their bio-applications [34,35]. Compared to thermal decomposition, traditional co-precipitation methods can obtain water soluble nanoparticles but of wide size distribution, and most of them have low crystallinity and therefore exhibit low stability [34,36,37,38]. 

In this study, ultra-small iron nanoparticles were synthesized by the co-precipitation method with some modification [39]. Polymethacrylic acid (PMAA), as an important water-soluble polymer ligand, has been widely used as a stabilizer for the synthesis of magnetic iron oxide nanoparticles. Its abundant carboxylic groups provide it with the ability to effectively coordinate with iron oxide nanoparticles surfaces [40,41]. Dodecanthiol (DDT) was used as a chain transfer agent to synthesize the water-soluble polymer ligand DDT-PMAA [34]. In order to make the nanoparticles uniform in size, the iron precursors were dissolved in concentrated HCl forming inert conditions to prevent their hydrolysis and condensation. The precipitating agents were added to the system by hot injection rapidly in a few seconds to separate the nucleation process and the growth process of NPs (nanoparticles) formation, leading to the formation of uniform NPs of high crystallinity due to the high temperature. The obtained magnetic iron nanoparticles were about 4.5 nm and had a narrow distribution. The cytotoxicity analysis of the magnetic iron oxide nanoparticles showed their biocompatibility even at a high concentration of 400 μg/mL using CCK-8 assay and no inflammation or tissue damage was found in the histology analysis. The high r_1_ relaxivity of the magnetic iron oxide nanoparticles (MIONs) make them attractive candidates for T_1_-positive contrast agents.

## 2. Materials and Methods

### 2.1. Reagents and Materials

Methacrylic acid (MAA, 99%), 2,2′-azobisisobutyronitrile (AIBN, 98%), anhydrous ethanol, Ferric chloride (FeCl_3_·6H_2_O, 99%), ferrous sulfate (FeSO_4_·7H_2_O, 99%), hydrochloric acid (HCl, 38%), ammonium hydroxide (NH_4_OH, 28%), anhydrous acetone, and anhydrous diethyl ether were purchased from National Medicines Corporation Ltd. of P. R. China (Beijing, China). Dodecanthiol (DDT, 99%) was purchased from Sigma-Aldrich (Shanghai, China). RPMI Medium 1640, Dulbecco’s modified eagle’s medium (DMEM), and phosphate buffer saline (PBS) were purchased from HyClone (Logan, UT, USA). Fetal bovine serum (FBS) was purchased from Gibco (Basel, Switzerland). Trypsin-EDTA (0.25%) phenol red, Penicillin-Streptomycin Solution, Cell Counting Kit (CCK-8) were purchased from Yeasen (Shang Hai, China).

### 2.2. Methods

#### 2.2.1. Preparation of Polymer Ligand DDT-PMAA

DDT-PMAA polymer ligand was synthesized using simple radical polymerization, DDT and PMAA acted as the transfer agent and the monomer, respectively. The length of polymer chain was adjusted by changing the molar ratio of DDT and PMAA. In a typical synthesis of polymer ligand DDT-PMAA, methacrylic acid (MAA, 5 g, 58 mmol), DDT (0.05 g, 0.29 mmol), 2,2′-azobisisobutyronitrile (AIBN, 0.095 g, 0.58 mmol), and 25 mL ethanol were mixed in a 100 mL four-necked flask with the magnetic stirring. The temperature was then maintained at 75 °C for 5 h. Subsequently, the mixture solution was cooled down to room temperature and the products were purified by cold diethyl either. The water-soluble polymer ligand DDT-PMAA was obtained after filtration using a Buchner funnel, and finally, the product was put into a vacuum at 45 °C to remove the surplus monomer, low polymer, and water.

#### 2.2.2. Preparation of MIONs@DDT-PMAA

The water soluble ultra-small iron oxide nanoparticles were prepared using the high-temperature co-precipitation method with some modification. In a typical synthesis of MIONs@DDT-PMAA, in order to maintain the oxygen-free environment, a three-necked flask with 50 mL twice purified water was inset in nitrogen for 30 min. In the presence of nitrogen, the water was heated to 80 °C and then the DDT-PMAA polymer ligand was added into the flask (1.5 mM, pH = 4). The iron precursor solution of 0.27 mM FeCl_3_·6H_2_O and 0.54 mM FeSO_4_·7H_2_O dissolved in 1 mL concentrated HCl was added to the flask, the color became yellow, and subsequently 15 mL NH_4_·H_2_O was injected rapidly. Then the color of the solution turned to black instantly. When the temperature approached 100 °C again, the reaction was allowed to proceed for 2 h at this temperature with vigorous magnetic stirring and constant nitrogen bubbling. Then the heating was stopped, and the reaction mixture was cooled down to room temperature under the nitrogen atmosphere. After that, the solvent was removed by rotary evaporation to a volume of nearly 50 mL and then the solution was dialyzed in deionized water for three days using dialysis membranes of molecular weight cut-off value 8 kD~14 kD, while changing the water at least three times. The dried black powder was obtained after rotary evaporation under the temperature of 45 °C that was placed in a vacuum oven maintained at 45 °C for two days.

#### 2.2.3. Characterization of MIONs@DDT-PMAA

To determine the morphology and size of MIONs@DDT-PMAA, transmission electron microscopy (TEM) was carried out with JEOL-2100 microscopes (Tokyo, Japan). The hydrodynamic diameters of MIONs in aqueous solution were studied using a dynamic light scattering (DLS, NanoZS90, Malvern, UK) [8]. The saturation magnetizations of the MIONs were recorded on a Lakeshore 7400 Series vibrating sample magnetometer (VSM) at 300K. X-ray diffraction (XRD) was obtained using D8 advance Bruker (Karlsruhe, Germany) in the 2θ range of 10°~90°. The X-ray photoelectron spectroscopy (XPS) was performed by PHI 5000C ESCA System (PHI, America). The Fourier transform infrared spectrum (FTIR) was conducted by Bruker Vertex 70 (Karlsruhe, Germany). Thermogravimetric analysis (TGA) measurements were made using TGA 8000 (Perkin Elmer, USA).

#### 2.2.4. Stability Test

The hydrodynamic size of MIONs@DDT-PMAA (1 mg/mL) was measured after dispersion in water of different pH values (3, 5, 7, 9, 11) and different NaCl salt concentrations (0, 0.5, 1.0, 1.5, 2.0, 2.5 M) solution after 24 h. Similarly, the stability profiles of the MIONs@DDT-PMAA were also studied in PBS and DMEM medium containing 10% fetal bovine serum (FBS) and 1% penicillin streptomycin (PS) [9]. The magnetic stability was obtained by monitoring the relaxation times over a period of seven days. The relaxivity time (T_1_ and T_2_) was also studied at different time points (4 h, 12 h, 24 h, 3 d, 7 d).

#### 2.2.5. Relaxivity Characterization and MR Imaging In Vitro

The saturation magnetization of the MIONs@DDT-PMAA were measured by varying the magnetic field between −10 and +10 kOe at 300 K. The longitudinal (T_1_) and transverse (T_2_) relaxation times were measured with a 1.41 T minispec mq 60 NMR Analyzer (Bruker, Karlsruhe, Germany) at 37 °C. Relaxivity values of r_1_ and r_2_ were calculated by fitting the 1/T_1_ and 1/T_2_ relaxation time (s^−1^) versus Fe concentration (mM) curves. The in vitro MR images were studied by a MesoMR23-060H-I MRI system (Niumag Corporation, Shanghai, China). The measurement conditions were as follows: T_1_-weighted sequence, multi-slice spin echo (MSE) TR/TE = 360/18 ms, FOV = 100 mm × 100 mm, thickness = 4.0 mm, 0.55 T, 32.0 °C. 

#### 2.2.6. In Vitro Cytotoxicity Assay

Cell Counting Kit-8 (CCK-8) assay was adopted to analyze the in vitro cytotoxicity of the obtained MIONs@DDT-PMAA. Cytotoxicity of the obtained MIONs@DDT-PMAA was evaluated with two kinds of cells (DC 2.4 and 4T1). Briefly, cells were incubated in a 96-well plate with 8 × 103 cells per well. Subsequently, the cells were incubated in the culture medium for 24 h at 37 °C and 5% CO_2_. After that, the existing DMEM medium containing 10% fetal bovine serum (FBS) and 1% penicillin streptomycin (PS) was replaced with fresh pure DMEM medium containing different concentrations of MIONs@DDT-PMAA. Then the cells were incubated for another 24 h and 48 h respectively, and then 10 μL CCK-8 solution was added into each well following 2 h of incubation. Later, OD value (A) of each well was determined by a microplate reader (Thermo, MULTISKAN MK3) at 450 nm. The following formula was used to calculate the viability of cell: cell viability (%) = [A (treatment group) − A (blank)]/[A (control) − A (blank)] × 100%.

#### 2.2.7. Animal Model and MR Imaging In Vivo

Animal procedures were in agreement with the guidelines of the Institutional Animal Care and Use Committee of Tongji University. The animal experiments were conducted on five-week-old female BALB/c mice. For establishment of tumor models, 2 × 106 4T1 cells suspended in 100 μL phosphate buffered saline (PBS) were subcutaneously injected into the right thigh of each mouse. For living imaging studies, BALB/c mice were anaesthetized by chloral hydrate via celiac injection before MR imaging.

Mouse MR imaging in vivo was performed on a 0.5 T MesoMR23-060H-I MR imaging system (Niumag Corporation, Shanghai, China) using a mouse (mouse mass ~20 g). MR images were obtained before and after tail intravenous injection with MIONs@DDT-PMAA at the dosage of 2.5 mg Fe per kg body weight at different intervals. The measured parameters were as follows: T_1_-weighted sequence, multi-slice spin echo (MSE) TR/TE = 420/18 ms, FOV = 100 mm × 100 mm, thickness = 2.5 mm, Slices = 8, average = 10, 0.55 T, 32.0 °C.

#### 2.2.8. In Vivo Biodistribution Analysis

For the biodistribution study, Balb/c mice (female, five weeks old) were intravenously injected with 150 μL MIONs@DDT-PMAA at the dosage of 20 mg/kg. Subsequently, the mice were sacrificed at different time points (5 h, 3 d, 14 d), and then the major organs (heart, liver, spleen, lung, kidney, and tumor) were collected and digested using 5 mL aqua regia overnight. The solutions were placed on a heating plate to volatilize the components and then the volume was made up to 10 mL using deionized water. The Fe concentration of the samples was quantified with ICP-MS [2].

#### 2.2.9. Histology Analysis

Hematoxylin and eosin (H&E) staining was used to test in vivo toxicity. The major organs (heart, liver, spleen, lung, kidney, and intestine) were removed 7 and 14 days after intravenous injection (2.5 mg Fe per kg body), and then fixed with paraformaldehyde (4%) sliced and stained with hematoxylin and eosin. Subsequently, the samples of organs were observed under an optical microscope (OLYMPUS CX43, Japan).

## 3. Results and Discussion

### 3.1. Synthesis and Characterization

**Scheme 1:**
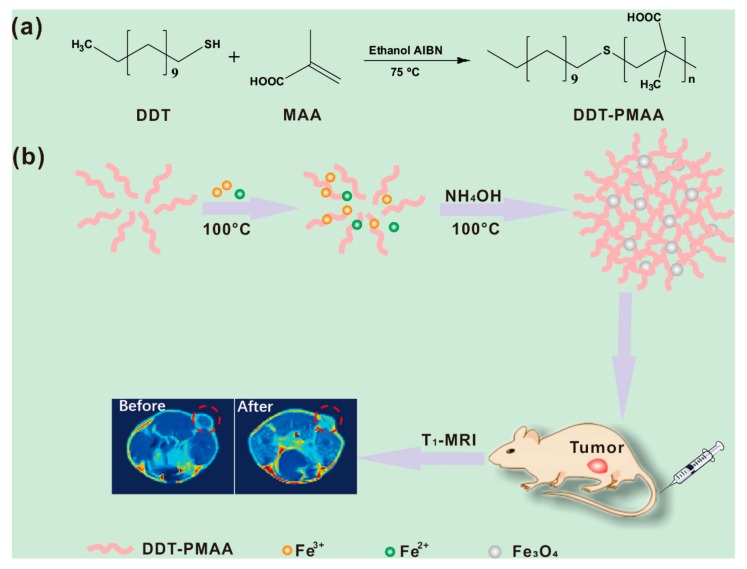
(**a**) Synthesis route of polymer ligand dodecanthiol-polymethacrylic acid (DDT-PMAA); (**b**) Schematic illustration of the synthesis route of MIONs@DDT-PMAA and its T_1_-weighted tumor MRI.

A series of polymer ligands of DDT-PMAA with four kinds of polymer chain length were synthesized by radical polymerization, DDT and PMAA acted as a transfer agent and monomer, respectively, as reported previously (Scheme 1a). Normalized GPC curves of DDT-PMAA synthesized using different DDT to monomer molar ratios are shown in Figure S1. We chose polymer ligand with molar ratios of 0.5:100 for subsequent experiments. The value of “*n*” in Scheme 1a was 168, the average molecular weight of (*M*_n_) was 14,624 g/mol, and the polydispersity (*M*_w_/*M*_n_) of the polymer ligand was 1.53. Scheme 1b illustrates the synthesis route of MIONs@DDT-PMAA using high-temperature co-precipitation method reported with some modification [38]. By co-precipitation of precursor solution containing FeCl_3_·6H_2_O and FeSO_4_·7H_2_O (molar ratio 2:1), MIONs were synthesized in the presence of ammonia with DDT-PMAA ligand. The rapid injection of precursors can lead to the formation of monodisperse nanoparticles (NPs), which causes an initial burst of nucleation at once followed by a quick growth of nuclei. The presence of HCl in the precursors can avoid the hydrolysis and condensation before the addition of precipitation agents. By using of polymer ligand of different concentration, the size, shape, and magnetic properties of the MIONs could be varied in a proper range [34].

**Figure 1:**
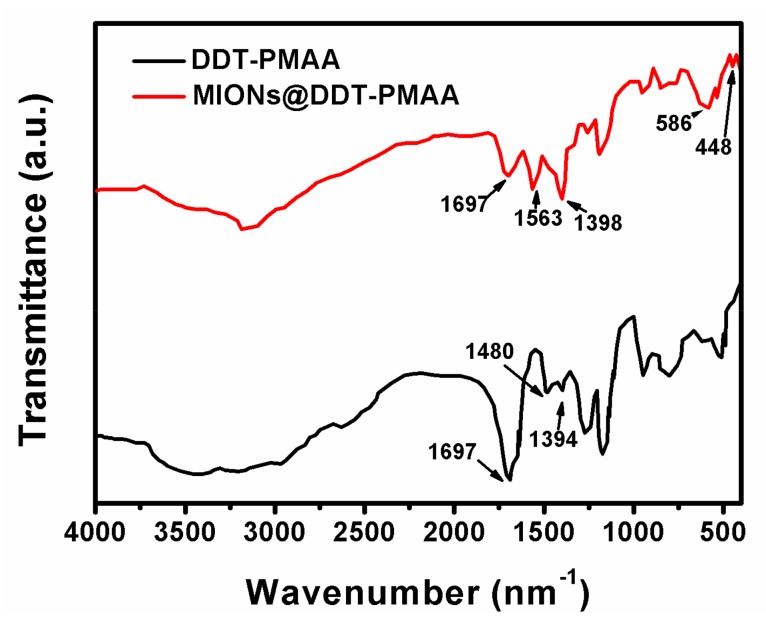
FTIR spectra of DDT-PMAA and MIONs@DDT-PMAA.

To determine that the polymer ligand DDT-PMAA was successfully linked to the surface of the MIONs, FTIR was conducted. As is shown in Figure 1, characteristic peaks appearing at 586 and 448 cm^−1^ confirms the existence of MIONS@DDT-PMAA, which are assigned to the torsion vibration and stretching of Fe–O bond of magnetite. The strong absorption peak at 1697 cm^−1^ corresponds to the carbonyl (–CO–) group, which could be observed both in DDT-PMAA and MIONs@DDT-PMAA. The symmetric and asymmetric stretching bands of carboxylate (–COO–) in polymer ligand DDT-PMAA are shifted from 1480, 1394 to 1563, and 1398 cm^−1^, respectively, it is possible that –COO– forms a coordination bond with Fe, which indicates that the polymer has bound to the surface of MIONS@DDT-PMAA. 

**Figure 2:**
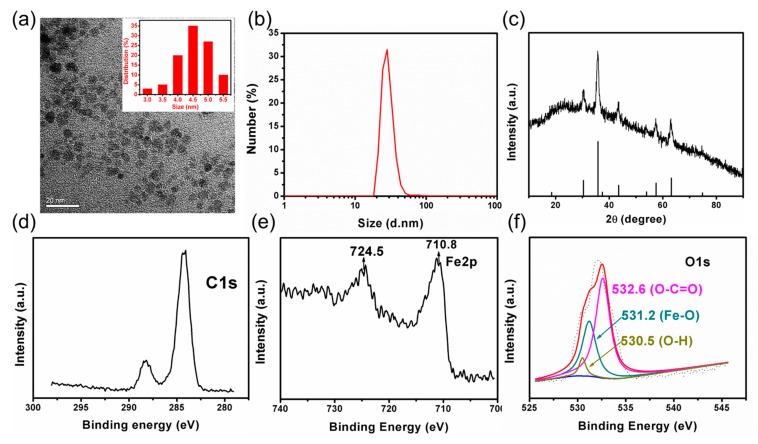
(**a**) TEM image and size distribution histogram of the MIONs@DDT-PMAA; (**b**) DLS data of MIONs@DDT-PMAA in water; (**c**) Diffractograms of MIONs@DDT-PMAA; (**d**–**f**) high-resolution C1s, Fe2p, O1s XPS spectra of the MIONs@DDT-PMAA.

The morphology and size of the MIONs@DDT-PMAA prepared with a series of DDT-PMAA ligands were characterized by TEM. Among them, the particles prepared with 2% DDT-PMAA at a concentration of 1.5 mM displayed a spherical or quasi-spherical shape with a quite uniform size distribution and we used it for subsequent experiments. The mean diameter of MIONs@DDT-PMAA was measured to be 4.5 ± 0.8 nm (Figure 2a), much smaller than the diameters measured by DLS (Figure 2b). TEM measures the size of single core particles, while DLS measures the hydrodynamic diameter comprising metal oxide core and water shell, this result is in agreement with previous literature reported [42]. 

The crystalline structure and phase of iron oxide in MIONs@DDT-PMAA was obtained by XRD analysis (Figure 2c). The peaks present at 2θ = 30.1°, 35.6°, 43.0°, 53.0°, 57.3°, and 62.9° correspond to (220), (311), (222), (400), (422), (511), and (440) reflections of magnetite (JCPDS: 75-0449), respectively, indicating the presence of crystalline spinel structured magnetite (Fe_3_O_4_). Figure S2 shows the full XPS spectra of the MIONs@DDT-PMAA. Figure 2d shows the C1s high-resolution XPS pattern, the actual measured C peak is 284.3 eV. Compared to the standard C peak (284.8 eV), the △ (positive charge correction value), which was used for calibration of other spectra was 0.5 eV. The Fe2p high resolution XPS pattern in Figure 1e reveals that the binding energy values of Fe2p3/2 and Fe2p1/2 are 710.8 and 724.5 eV, respectively, which is also consistent with the literature reports [43]. The O1s spectrum (Figure 1f) of Fe_3_O_4_ nanoparticles displays three peaks at 530.5, 531.2, and 532.6, which can be assigned to oxygen atoms of O–H bonds, Fe–O bonds, and O–C=O groups, respectively, thus demonstrating the presence of the carboxylate groups on the surface of the Fe_3_O_4_ nanoparticles. To further confirm the success coating, TGA analysis was conducted (Figure S3). The polymer ligand DDT-PMAA was almost completely decomposed at 540 °C. From TGA analysis, it can be concluded that the polymer wt % in the MIONs@DDTPMAA was 77% and iron oxide wt % was 23%. The successful coating of the polymer ligand can also be confirmed by Zeta potential analysis; the surface potential comes to −60 mV due to the carboxylic groups (–COOH) of PMAA.

### 3.2. Stability Study of the MIONs@DDT-PMAA

**Figure 3:**
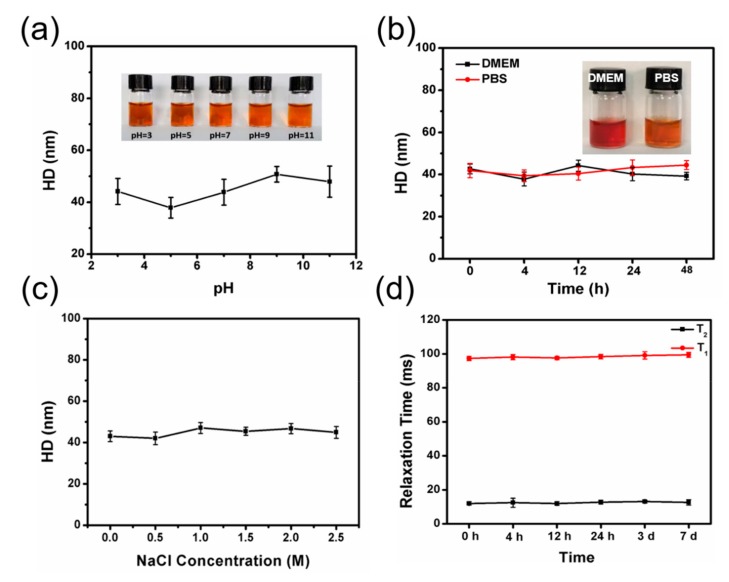
(**a**) Digital photographic images and hydrodynamic diameters of MIONs@DDT-PMAA at different pH; (**b**) Digital photographic images and hydrodynamic diameters of MIONs@DDT-PMAA at PBS and Dulbecco’s modified eagle medium (DMEM) at different times; (**c**) Hydrodynamic diameters in different NaCl concentrations; (**d**) The longitudinal (T_1_) and the transverse (T_2_) time at different time points.

The colloidal stability of MIONs@DDT-PMAA was investigated by incubating them with different media/solutions i.e., deionized water, phosphate-buffered saline (PBS), Dulbecco’s modified eagle medium (DMEM), and NaCl salt solution. Hydrodynamic diameter and relaxation time were monitored as the key criteria to assess their colloidal stability. The hydrodynamic diameter was determined in the different pH of aqueous solution (Figure 3a). The hydrodynamic diameter was almost unchanged, and no macroscopic aggregates were observed in a broad range of pH (3–11), as shown in the digital photographs. As shown in Figure 3b, MIONs@DDT-PMAA could easily be dissolved in PBS and DMEM (containing 10% FBS and 1% PS) with no aggregation, and meanwhile the size did not change a lot within two days. Similarly, the hydrodynamic diameter in different NaCl solutions did not change a lot (Figure 2c). The relaxation time (T_1_ and T_2_) of MIONs@DDT-PMAA also maintained a stable level (Figure 2d) for at least seven days.

### 3.3. Magnetic Properties Characterization and In Vitro MRI

**Figure 4:**
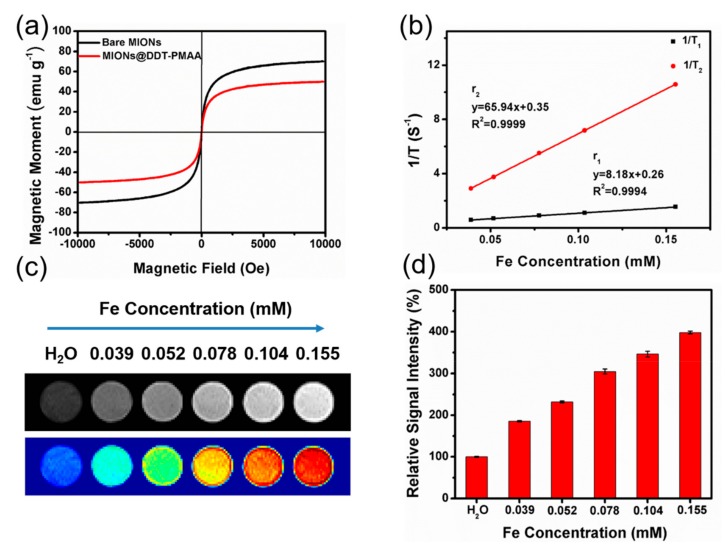
(**a**) Magnetization curves of MIONs@DDT-PMAA; (**b**) T_1_ relaxation rates (1/T_1_) and T_2_ relaxation rates (1/T_2_) of MIONs@DDT-PMAA; (**c**) T_1_-weighted images of MIONs@DDT-PMAA in different Fe concentrations; (**d**) Corresponding relative signal intensities in different Fe concentrations.

The magnetization curves of the MIONs@DDT-PMAA were measured by varying the magnetic field between −10 and +10 kOe. As shown in Figure 4a, the saturation magnetization was 49.5 emu/g without any evident remanence or coercivity and was lower than the bare MIONs (70 emu/g) but still exhibited great properties. MR contrast agents were evaluated on the basis of their relaxivity or change in the relaxation rates of water protons in the presence of the contrast agent per unit concentration. The MRI properties of the MIONs@DDT-PMAA were evaluated using a 1.41T MRI facility at 37 °C in water by measuring the longitudinal (r_1_) and the transverse (r_2_) nuclear magnetic relaxation rates of water protons. MIONs@DDT-PMAA have high r_1_ values of 8.18 mM^−1^·S^−1^ and a remarkably low r_2_/r_1_ value of 8.08 that suggest their ideal candidacy for T_1_-weighted MR contrast agents (Figure 4b). Figure 4c shows T_1_-weighted images of MIONs@DDT-PMAA in vitro at different Fe concentrations. It is obvious that signal intensity is enhanced with an increase in the concentration of Fe in samples, which is further demonstrated intuitively in Figure 4d. Their high r_1_ value and excellent MRI effect show that these MIONs are promising candidates as T_1_-weighted MR contrast agents.

### 3.4. In Vitro Toxicity Test

**Figure 5:**
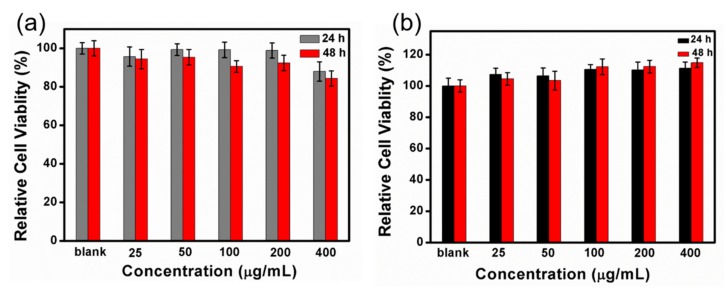
Cytotoxicity of MIONs@DDT-PMAA in different concentrations of DC 2.4 cells (**a**) and 4T1 cells (**b**) determined by the CCK-8 cell viability assay.

Before the use of the MIONs@DDT-PMAA for in vivo MR imaging applications, the cytotoxicity of the particles was evaluated by the CCK-8 cell viability assay. Cytotoxicity of MIONs@DDT-PMAA at different concentrations and time points was evaluated on DC 2.4 cells using the CCK-8 assay (Figure 5a). The cell viability turns out to be more than 80% at all investigated concentrations. Even at the highest concentration of 400 μg/mL, MIONs@DDT-PMAA exhibit negligible toxicity, 88% and 84% of the DC 2.4 cells survive relative to the control group after incubation with MIONs@DDT-PMAA for 24 and 48 h, respectively. These cytotoxicity data indicate that these nanoparticles are suitable for biological applications. The cytotoxicity test was also conducted in 4T1 cells, and the results show even a slightly better growth of cells after incubation with MIONs@DDT-PMAA (Figure 5b) again, demonstrating their biocompatibility.

### 3.5. In Vivo MRI Study

**Figure 6:**
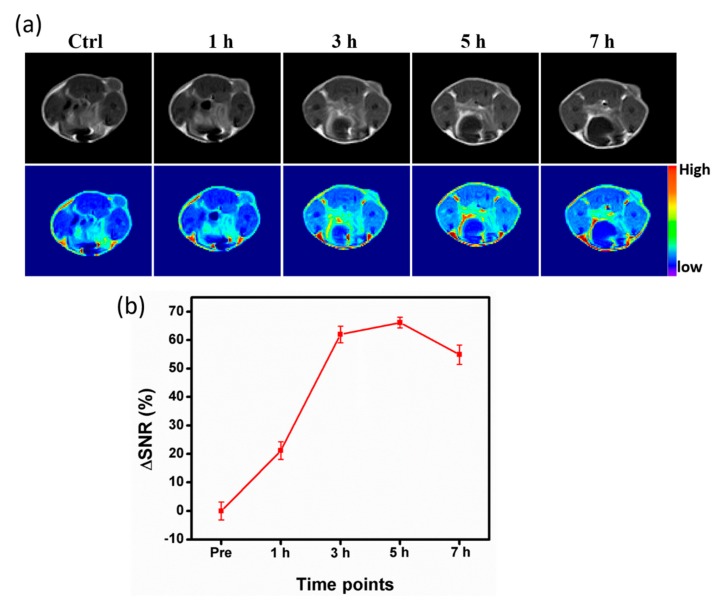
Mouse tumor T_1_-weighted MRI images (**a**) and relative MR signal-to-noise ratio (**b**) at different time points before and after intravenous injections of MIONs@DDT-PMAA at the dosage of 2.5 mg Fe per kg body weight.

In vivo MR imaging on 4T1 tumor-bearing mice was subsequently conducted. After an intravenous injection of MIONs@DDT-PMAA (dosage: 2.5 mg Fe/kg mice), the T_1_-weighted contrast was obtained from 0 to 7 h (Figure 6a). These results show that the tumor started brightening within 1 h and maintained a high level to 7 h. To quantify the contrast enhancement, we calculated the signal-to-noise ratio (SNR) by finely analyzing regions of interest (ROIs) of the images and defined the contrast enhancement as the increase of SNR, △SNR = (|SNRpost − SNRpre|)/SNRpre (Figure 6b). The contrast enhancement indicated the accumulation of MIONs@DDT-PMAA in the tumor. These results show that the MIONs@DDT-PMAA are highly promising candidates for the MRI positive imaging.

### 3.6. Biodistribution Study and Histology Toxicity Analysis

**Figure 7:**
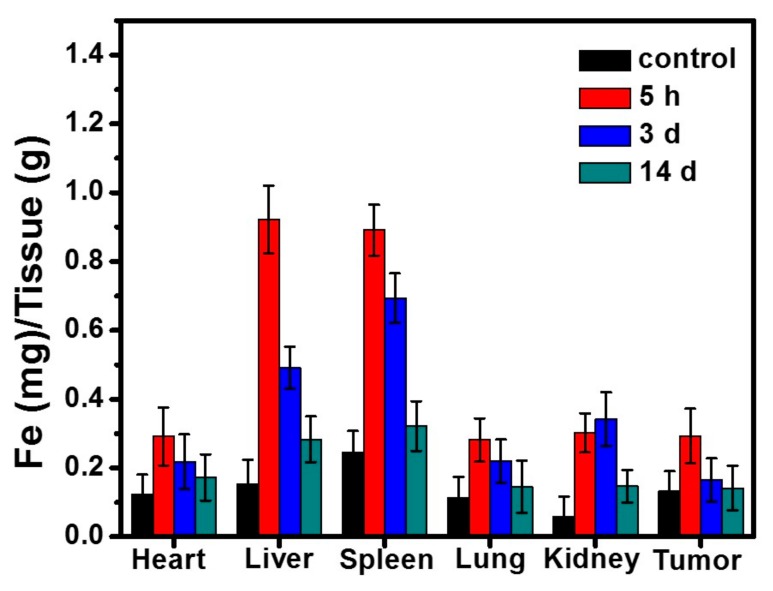
Time-dependent biodistribution measurement of Fe levels in various organs of mice after intravenous injection of MIONs@DDT-PMAA.

To evaluate the in vivo clearance process and organ distribution, mice were injected with the MIONs@DDT-PMAA nanoparticles via intravenous injection (dosage: 20mg/kg mice) and then sacrificed at different time points. The main organs were removed and dissolved in digesting chloroazotic acid to analyze the total amount of Fe in blood using ICP-MS. As shown in Figure 7, a large quantity of MIONs@DDT-PMAA was accumulated in organs of the reticuloendothelial system, such as the liver and spleen. At 5 h, the nanoparticles were clustered in the tumor and corresponded to the enhanced in vivo MRI contrast signals. After 3 and 14 days, the quantity of the nanoparticles in all organs was found to be decreased, which shows their successful clearance from the body.

**Figure 8:**
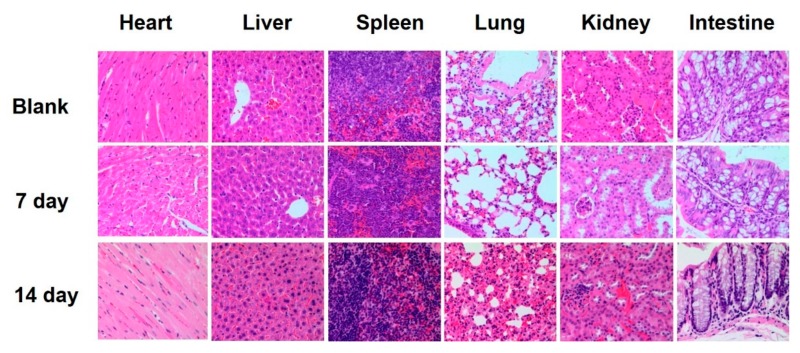
Hematoxylin and eosin-stained tissue sections from mouse receiving no injection and injected with MIONs@DDT-PMAA for 7 and 14 days.

Apart from the cytotoxicity test, in vivo toxicity of MIONs@DDT-PMAA was further investigated with H & E staining examination (Figure 8). The mice were sacrificed, and the major organs were collected and treated to evaluate the histological changes. All investigated organs of the experimental mouse were found normal compared to the control group. No necrosis or inflammatory infiltrate was found in any of the groups. It can be inferred from in vitro cytotoxicity and the in vivo histological study that MIONs@DDT-PMAA are biocompatible.

## 4. Conclusions

In summary, we report a convenient protocol to prepare highly stable DDT-PMAA polymer ligand functionalized ultra-small Fe_3_O_4_ nanoparticles for T_1_-weighted positive MR imaging of tumors in vivo. MIONs@DDT-PMAA synthesized by a modified co-precipitation method have a uniform size of ca. 4.5 nm. These NPs can easily disperse in water and have good biocompatibility and colloidal stability. Due to the high crystallinity, they have great magnetic properties with an r_1_ relaxation rate of 8.18 mM^−1^·S^−1^. T_1_-weighted MRI was also successfully performed using these nanoparticles. The rich chemistry of polymer at the surface of the nanoparticles offers their easy surface engineering to create a multitude of functional groups at their surface to make them promising candidates for a host of biological applications.

## Supplementary Materials

The supplementary materials are available online at https://www.mdpi.com/2073-4360/11/5/882/s1.
