# Supplementary material for: Fe3O4 Nanoparticles Functionalized with Polymer Ligand for T1-Weighted MRI In Vitro and In Vivo
Source: Polymers (Basel). 2019 May 14;11(5):882. doi: 10.3390/polym11050882 (PMC6572598; doi:10.3390/polym11050882)
Supplement: Supplementary file 1 [file polymers-11-00882-s001.pdf]

Supporting Information

Fe_3O_4 Nanoparticles Functionalized with Polymer Ligand for T_1-weighted MRI In Vitro and In Vivo

Chenyang Xiang [1, 2, 3], Xin Zhong [1], Weitao Yang [2], Muhammad Irfan Majeed [3], Jun Wang [2], Jiani Yu [2], Jinming Hu [4], Zushun Xu [1], Bien Tan [3] *, Bingbo Zhang [2] *, Wei Yan [1, 3] *

[1] Hubei Collaborative Innovation Center for Advanced Organic Chemical Materials, Ministry of Education, Key Laboratory of Green Preparation and Application for Functional Materials, Hubei Key Laboratory of Polymer Materials, School of Materials Science & Engineering, Hubei University, Wuhan 430062, China; willieyancn2003@aliyun.com (W. Yan)

[2] The Institute for Biomedical Engineering & Nano Science, Tongji University School of Medicine, Shanghai 200443, China; bingbozhang@tongji.edu.cn (B. Zhang)

[3] School of Chemistry and Chemical Engineering, Huazhong University of Science and Technology, Wuhan 430074, China; bien.tan@mail.hust.edu.cn (B. Tan)

[4] CAS Key Laboratory of Soft Matter Chemistry, Department of polymer Science and Engineering, University of Science and Technology of China, Hefei, Anhui 230026, China; jmhu@ustc.edu.cn

* Correspondence: willieyancn2003@aliyun.com (W.Yan); Tel.: 02788661729, bingbozhang@tongji.edu.cn (B. Zhang); Tel.: + 86 21 65988029, bien.tan@mail.hust.edu.cn (B. Tan); Tel.: 02787558172

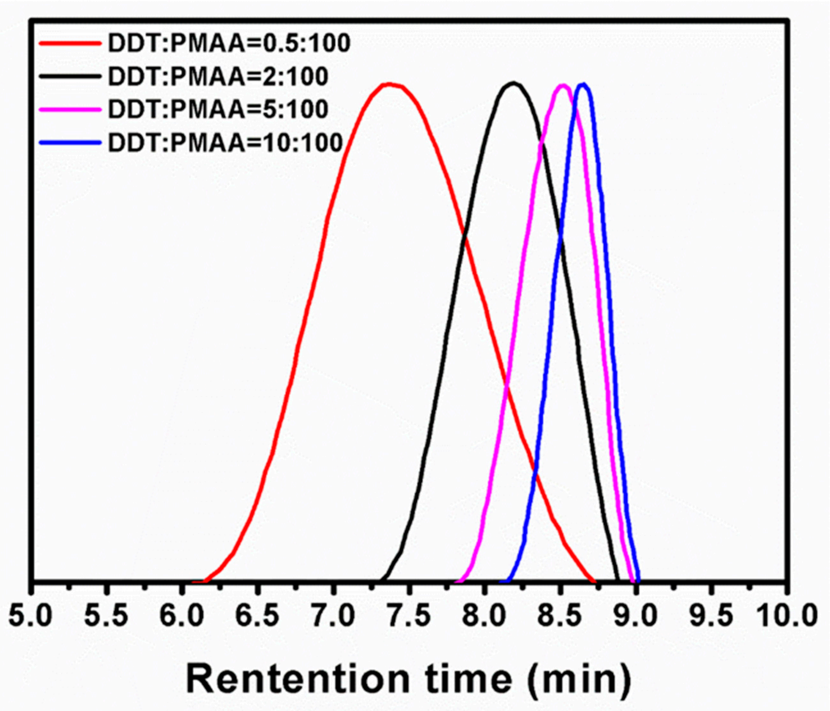

Figure S1. Normalized GPC curves of DDT-PMAA synthesized using different DDT to monomer molar ratios.

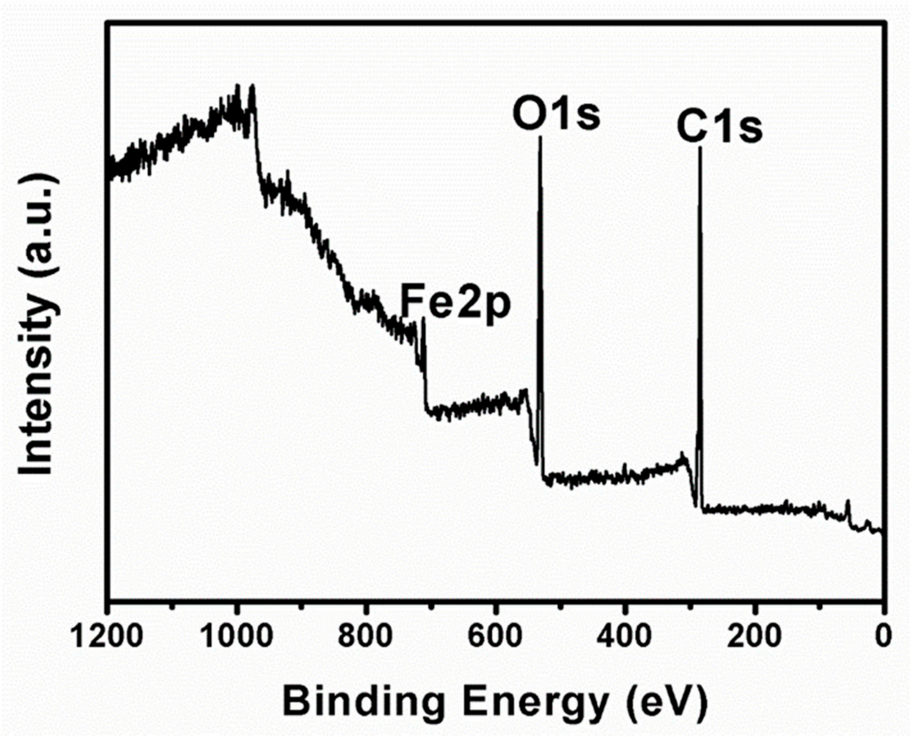

Figure S2. XPS full spectrum of MIONs@DDT-PMAA.

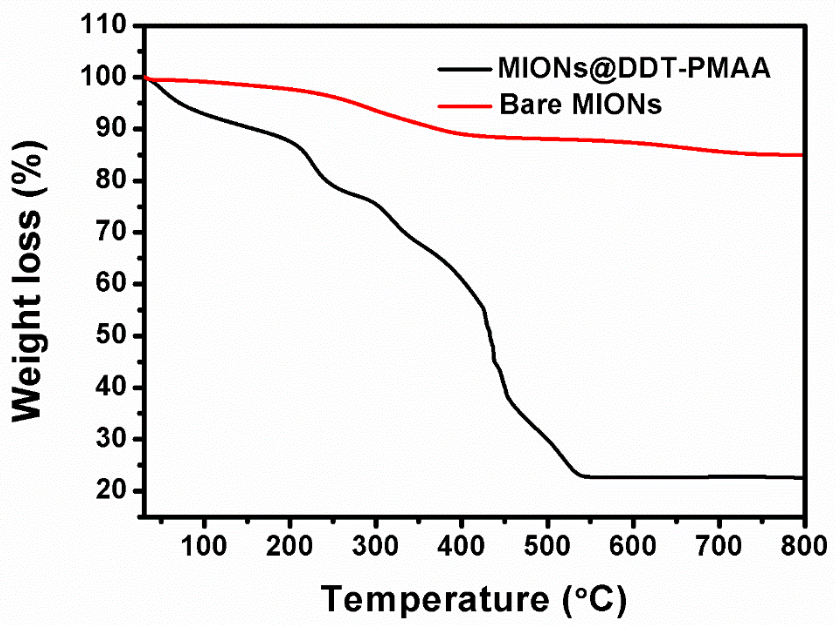

Figure S3. TGA analysis of MIONs@DDT-PMAA.
